# Immunonutritive Scoring in Patients With Hepatocellular Carcinoma Undergoing Transarterial Chemoembolization: Prognostic Nutritional Index or Controlling Nutritional Status Score?

**DOI:** 10.3389/fonc.2021.696183

**Published:** 2021-06-10

**Authors:** Lukas Müller, Felix Hahn, Aline Mähringer-Kunz, Fabian Stoehr, Simon J. Gairing, Friedrich Foerster, Arndt Weinmann, Peter R. Galle, Jens Mittler, Daniel Pinto dos Santos, Michael B. Pitton, Christoph Düber, Roman Kloeckner

**Affiliations:** ^1^ Department of Diagnostic and Interventional Radiology, University Medical Center of the Johannes Gutenberg University Mainz, Mainz, Germany; ^2^ Department of Internal Medicine I, University Medical Center of the Johannes Gutenberg University Mainz, Mainz, Germany; ^3^ Department of General, Visceral and Transplant Surgery, University Medical Center of the Johannes Gutenberg University Mainz, Mainz, Germany; ^4^ Department of Radiology, University Hospital of Cologne, Cologne, Germany

**Keywords:** hepatocellular carcinoma, transarterial chemoembolization, immunonutritive scoring, prognostic nutritional index, controlling nutritional status, survival prediction, risk scoring

## Abstract

**Objectives:**

The Prognostic Nutritional Index (PNI) and Controlling Nutritional Status (CONUT) score are immunonutritive scoring systems with proven predictive ability in various cancer entities, including hepatocellular carcinoma (HCC). We performed the first evaluation of the CONUT score for patients undergoing transarterial chemoembolization (TACE) and compared CONUT and PNI in the ability to predict median overall survival (OS).

**Methods:**

Between 2010 and 2020, we retrospectively identified 237 treatment-naïve patients with HCC who underwent initial TACE at our institution. Both scores include the albumin level and total lymphocyte count. The CONUT additionally includes the cholesterol level. Both scores were compared in univariate and multivariate regression analyses taking into account established risk factors. In a second step, a subgroup analysis was performed on BCLC stage B patients, for whom TACE is the recommended first-line treatment.

**Results:**

A high CONUT score and low PNI were associated with impaired median OS (8.7 *vs*. 22.3 months, p<0.001 and 6.8 *vs*. 20.1 months, p<0.001, respectively). In multivariate analysis, only the PNI remained an independent prognostic predictor (p=0.003), whereas the CONUT score lost its predictive ability (p=0.201). In the subgroup of recommended TACE candidates, both CONUT and PNI were able to stratify patients according to their median OS (6.6 *vs*. 17.9 months, p<0.001 and 10.3 *vs*. 22.0 months, p<0.001, respectively). Again, in the multivariate analysis, only the PNI remained an independent prognostic factor (p=0.012).

**Conclusion:**

Both scores were able to stratify patients according to their median OS, but only the PNI remained an independent prognostic factor. Therefore, PNI should be preferred when evaluating the nutritional status of patients undergoing TACE.

## Introduction

Hepatocellular carcinoma (HCC) is one of the most common cancers and among the deadliest (1, 2). According to the European Association for the Study of the Liver (EASL) and the American Association for the Study of Liver Diseases (AASLD) guidelines, the Barcelona Clinic Liver Cancer (BCLC) classification system is the preferred framework for predicting prognosis and allocating treatment (3, 4). Following these suggestions, transarterial chemoembolization (TACE) is the standard of care for patients with intermediate-stage HCC (5, 6). However, in clinical reality, this intermediate stage comprises a heterogeneous group of patients with considerable differences in tumor burden and liver function (7–10). Thus, prognosis prediction and treatment decision-making remain difficult in these patients. Several scoring systems have been proposed to help clinicians (11–13) but have all failed external validation, creating a need for novel attempts and biomarkers (14, 15).

One promising approach may be the inclusion of growing knowledge on the influence of inflammation on tumor development and progression (16, 17), an aspect that is currently underrepresented in available suggestions for TACE (3, 4).

One possibility for translating the observed preclinical results in daily clinical routine is the Prognostic Nutritional Index (PNI), which combines the lymphocyte count with the albumin level as an indicator of the nutritional status of patients (18). Originally derived in 1980 by Buzby et al. for patients undergoing gastrointestinal surgery (19), the PNI has been identified as an independent prognostic factor for various cancer entities (18) and is experiencing a renaissance in cancer research.

Particularly for patients with HCC, the PNI may be a powerful prognostic tool, as it partly reflects the complex combination of cancer in chronic liver inflammation processes, leading to impaired organ function. This hypothesis is supported by the first promising results in patients with HCC, in which the PNI was identified as a prognostic factor for median overall survival (OS) (20–22). Recently, Liu et al. and He et al. were the first to show an influence on the median OS for patients undergoing TACE (23, 24). Both studies were performed using data based mainly on Asian patients, and an evaluation of the PNI in Western TACE patients is still lacking.

In addition to the PNI, a novel promising immunonutritive score is available: the Controlling Nutritional Status (CONUT) (25, 26). The CONUT includes the cholesterol level in addition to albumin and lymphocyte count. This score has been highly predictive in various cancer entities (27–29). For HCC, the CONUT score has been evaluated mainly in patients undergoing surgery (30, 31), and it has never been investigated in patients undergoing TACE. Furthermore, a comparison of both immunonutritive scoring systems is lacking.

The purpose of the present study was to perform an external validation of the PNI and CONUT scores for patients with HCC undergoing TACE and to compare them in terms of prognostic power.

## Materials and Methods

This study was approved by the responsible ethical body (Ethics committee of the Medical Association of Rhineland Palatinate, Mainz, Germany) for the retrospective analysis of clinical data (permit number 2021-15666). The requirement for informed consent was waived. Patient records and information were anonymized and de-identified prior to analysis. TRIPOD guidelines were followed for the writing process.

### Patients

**Figure 1:**
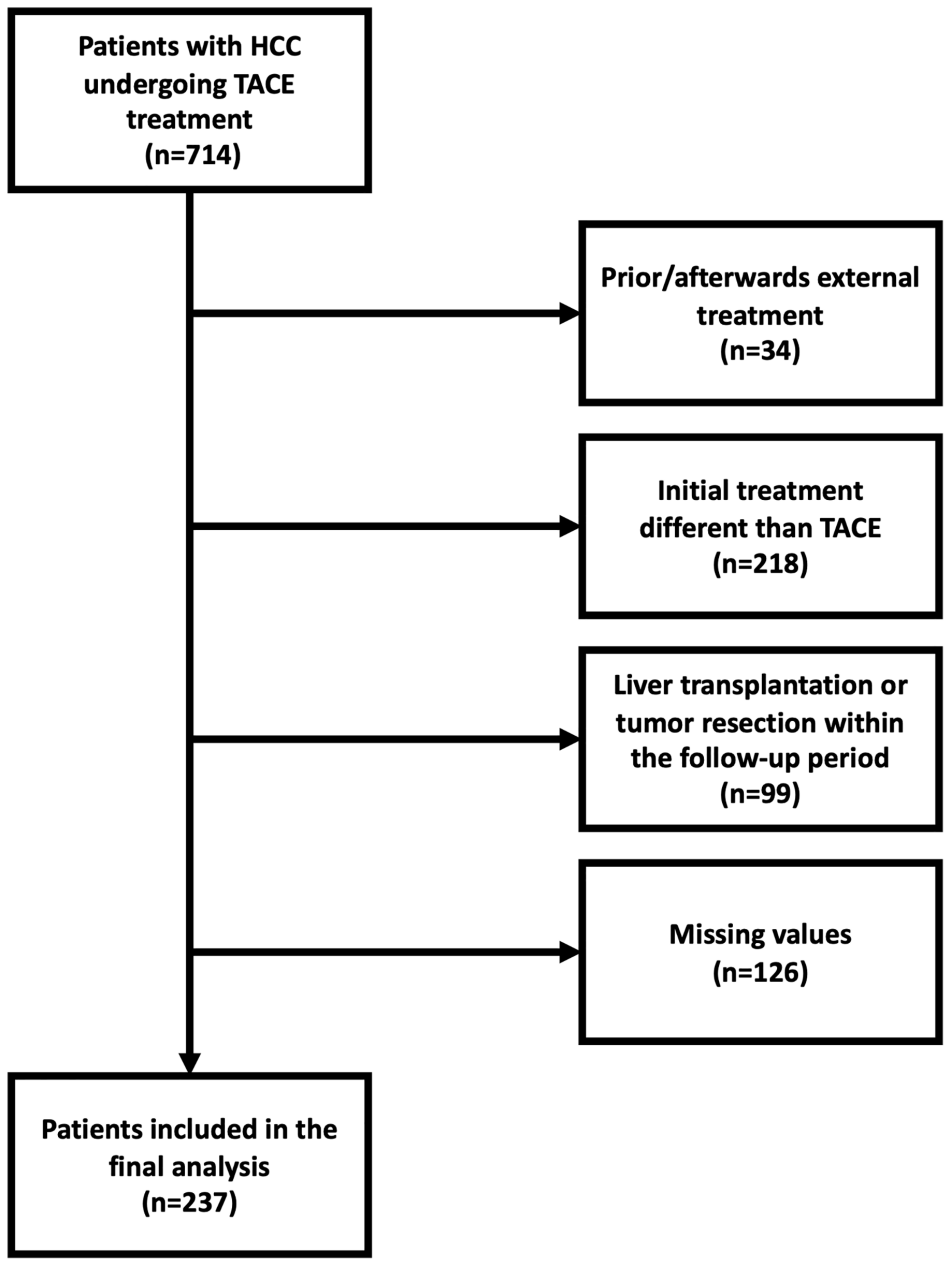
Flowchart providing the reasons for dropouts and the final number of patients for whom the PNI and CONUT score could be evaluated. HCC, hepatocellular carcinoma; TACE, transarterial chemoembolization.

Between January 2010 and November 2020, a total of 714 patients with confirmed HCC were referred to our tertiary care center for TACE. For the reasons shown in **Figure 1**, 477 of these patients had to be excluded. Thus, 237 patients were included in the final analysis. A subgroup analysis was performed with 126 (53.2%) BCLC stage B patients, for whom TACE treatment is the recommended first-line therapy according to current recommendations (3, 32).

### Diagnosis, Treatment, and Follow-Up

HCC was diagnosed using histological or image-derived EASL criteria (3). All patients underwent contrast-enhanced CT or MRI prior to their first TACE treatment. Follow-up comprised clinical examination, blood sampling, and cross-sectional imaging, which was typically repeated every 6 weeks in the case of a viable tumor. In the case of a complete response, this interval was extended to 12 weeks. Prior to each treatment decision, all patients underwent an extensive discussion in an interdisciplinary tumor board consisting of hepatologists/oncologists, diagnostic and interventional radiologists, visceral surgeons, pathologists, and radiation therapists. TACE was performed in a standardized manner as described in detail elsewhere (33, 34). The primary endpoint was OS, which was defined as the time interval between the initial TACE session and death or last follow-up.

### Data Acquisition

The dataset was acquired from the clinical registry unit (CRU). The CRU is an established registry that prospectively collects all patients with liver cancer treated at our tertiary referral center (35). The CRU dataset includes all baseline characteristics, including demographic data, liver disease status and etiology, laboratory parameters, TACE-related parameters, and information on the tumor burden, including tumor growth pattern, number of lesions, and the diameter of the largest target lesion. In case of missing data, this information was updated using the radiology information system and the laboratory database.

### Calculation of PNI and CONUT

**Figure 2:**
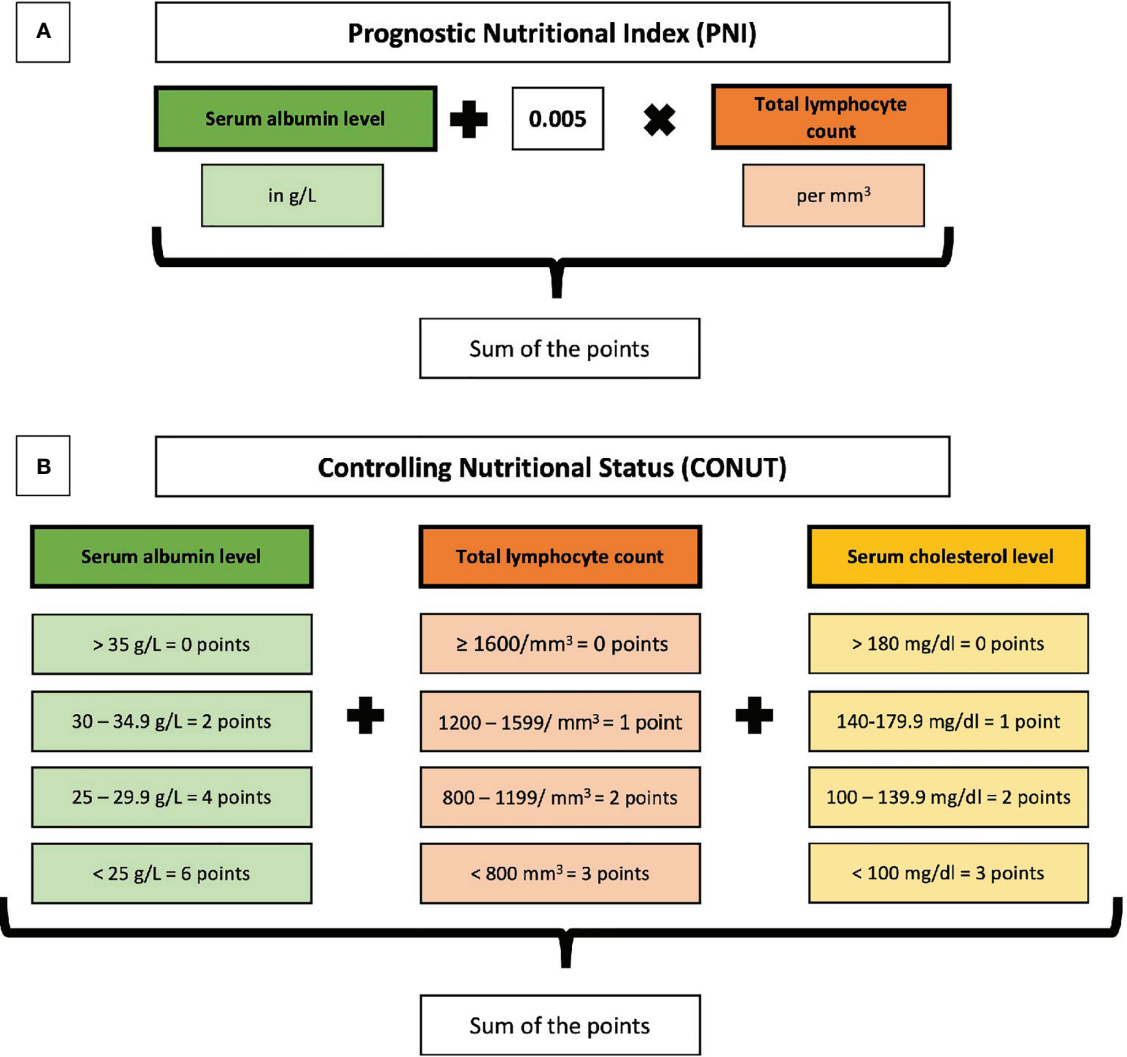
Overview of the calculation and parameters included in the Prognostic Nutritional Index (PNI) **(A)** and the Controlling Nutritional Status (CONUT) **(B)**.

PNI and CONUT were calculated as described in the original publications (19, 25). The parameters included and their weights are shown in **Figure 2**.

### Statistical Analysis

Statistical analysis and graphic design were performed in R 4.0.3 (A Language and Environment for Statistical Computing, R Foundation for Statistical Computing, http://www.R-project.org; accessed 2021). Categorical and binary baseline parameters were reported as absolute numbers and percentages. Continuous data were reported as median and range. Standardized cut-offs for the laboratory parameters were derived from our laboratory database. The PNI and CONUT cut-off values were calculated using optimal stratification with the packages “survminer” and “survival” (https://cran.r-project.org/package=survminer https://cran.r-project.org/package=survminer, https://CRAN.R-project.org/package=survival, accessed 2021). The same packages were used to perform survival analysis, creating Kaplan–Meier curves and strata compared by log-rank testing. Multivariate Cox proportional hazards regression models assessing hazard ratios (HRs) and corresponding 95% confidence intervals (CIs) were used to determine the effect of the risk stratification and to evaluate the roles of included factors. A p-value of <0.05 was considered statistically significant for all tests.

## Results

### Baseline Characteristics

The baseline characteristics at initial TACE treatment are presented in **Table 1**. Subgroup analysis was performed on 126 patents with BCLC stage B (i.e., the recommended TACE subgroup).

**Table 1:** Baseline characteristics of patients with HCC undergoing TACE.

Variable	All patients(n=237)	Recommended TACE subgroup (n=126)
Median age, years (IQR)	70 (62-75)	70 (63-75)
Gender, n (%)		
Female	197 (16.9)	18 (14.3)
Male	40 (83.1)	108 (85.7)
Etiology, n		
Alcoholic	112	57
Hepatitis C	38	19
Hepatitis B	24	16
NASH	23	
Hemochromatosis	5	3
AIH/PBC/PSC	4	3
Unknown/Other	23	11
Child-Pugh stage, n (%)		
A	94 (39.7)	54 (42.9)
B	94 (39.7)	57 (45.2)
C	21 (8.8)	0
No cirrhosis	28 (11.8)	15 (11.9)
BCLC stage, n (%)		
0	0	0
A	43 (18.1)	0
B	126 (53.2)	126 (100.0)
C	48 (20.3)	0
D	20 (8.4)	0
Median max. tumor size, cm (IQR)	4.1 (2.8-6.2)	4.2 (3.0-5.9)
Tumor number, n (%)		
Unifocal	55 (23.2)	0
Multifocal	167 (70.5)	120 (95.2)
Diffuse growth pattern, n (%)	15 (6.3)	6 (4.8)
Median albumin level, g/L (IQR)	32 (28-36)	33 (29-36)
Median lymphocyte count, per mm^3^ (IQR)	1221 (831-1599)	1279 (841-1743)
Median cholesterol level, mg/dl (IQR)	165 (137-203)	167 (138-213)
Median bilirubin level, mg/dl (IQR)	1.3 (0.8-2.1)	1.2 (0.8-1.9)
Median platelet count, per nl (IQR)	128 (86-193)	123 (82-188)
Median AST level, U/L (IQR)	63 (45-97)	62 (47-87)
Median ALT level, U/L (IQR)	42 (28-62)	41 (27-61)
Median INR (IQR)	1.2 (1.1-1.3)	1.1 (1.0-1.3)
Median AFP level, ng/ml (IQR)	44 (8.5-744.5)	47 (8.8-536.3)
Type of TACE		
cTACE	82 (34.6)	35 (27.8)
DEB-TACE	155 (65.4)	91 (72.2)

### Survival Analysis

**Figure 3:**
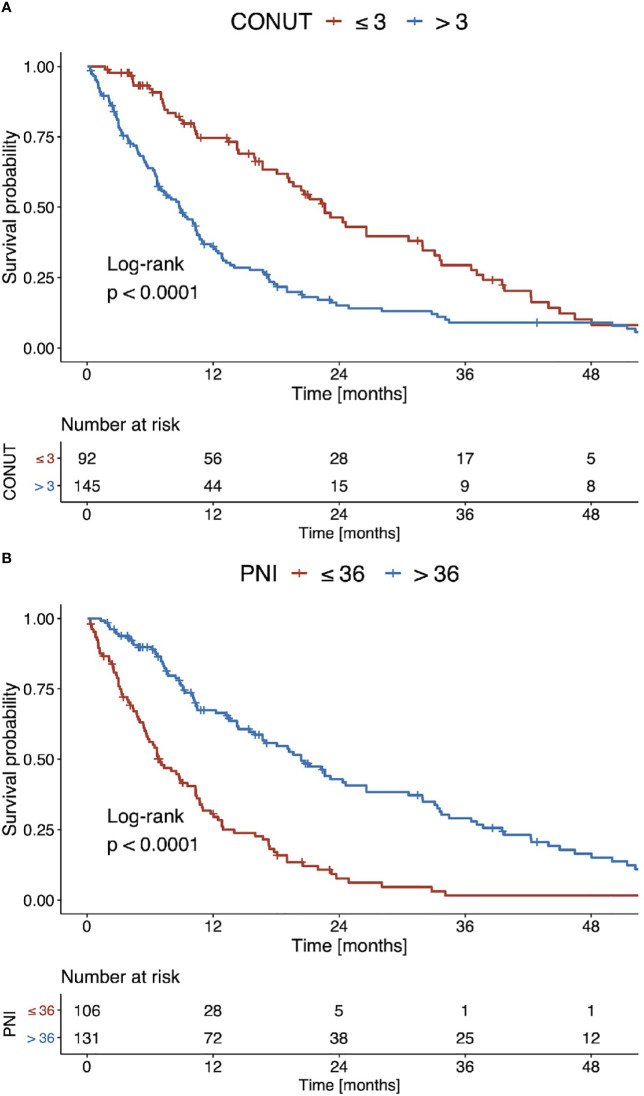
Kaplan Meier curves of overall survival stratified according to **(A)** the CONUT score and **(B)** the PNI for the entire patient cohort.

Using optimal stratification for median OS, the best cut-off for the CONUT score was 3 points. Using this cut-off, 145 (61.2%) patients had a high CONUT score and 92 (38.8%) a low score. The corresponding median OS for high and low CONUT scores was 8.7 months and 22.3 months (p<0.001, **Figure 3A**), respectively. The best cut-off for the PNI was 36 points. Using this cut-off, 131 (55.3%) patients had a high PNI and 106 (44.7%) a low PNI. The median OS for low and high PNI values was 6.8 months and 20.1 months (p<0.001, **Figure 3B**), respectively.

**Figure 4:**
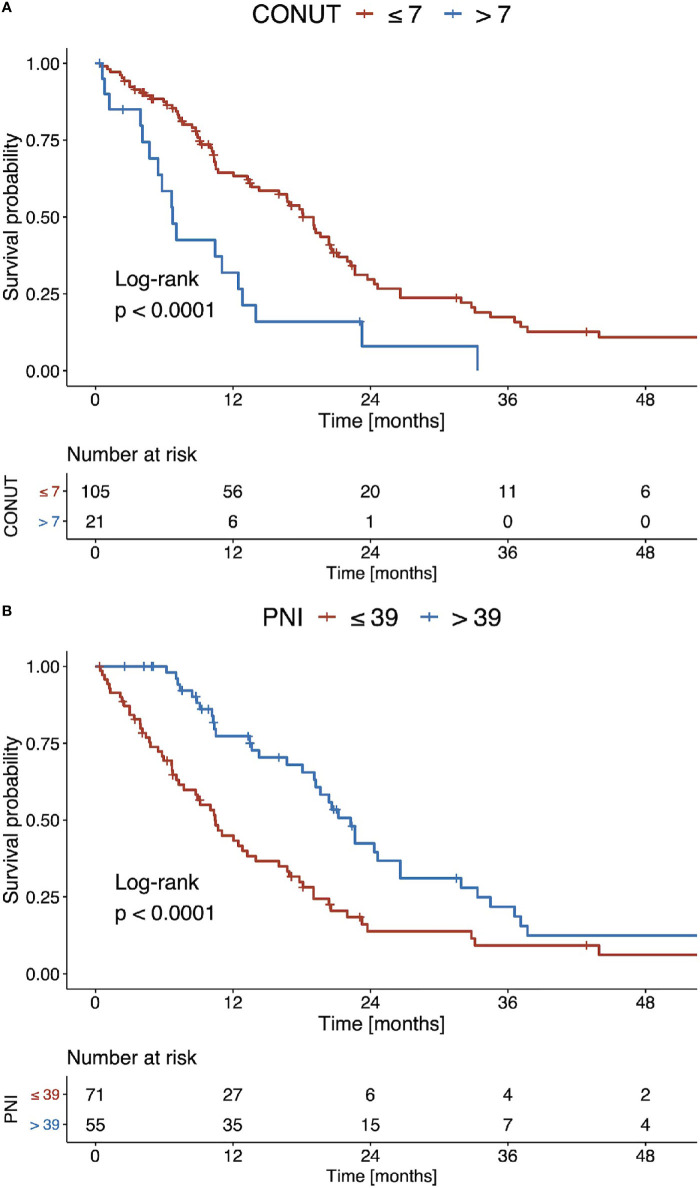
Kaplan Meier curves of overall survival stratified according to **(A)** the CONUT score and **(B)** the PNI for the recommended TACE candidates (BCLC B).

Using optimal stratification for median OS, the best cut-off for the CONUT score was 7 points. Using this cut-off, 21 (16.7%) patients had a high CONUT score and 105 (83.3%) a low score. The corresponding median OS for high and low CONUT scores was 6.6 months and 17.9 months, respectively (p<0.001, **Figure 4A**). The best cut-off for the PNI was 39 points. Using this cut-off, 55 (43.7%) patients had a high PNI value and 71 (56.3%) a low PNI value. The median OS for low and high PNI values was 10.3 months and 22.0 months, respectively (p<0.001, **Figure 4B**).

Univariate Cox hazard regression indicated a high prognostic value for both the CONUT score and the PNI, as well as the bilirubin level. None of the other included risk factors reached significance. In the subsequent multivariate analysis, including all of the above-mentioned factors, only a low PNI and high bilirubin levels remained significant as predictors (**Table 2**), as the CONUT score lost its predictive ability.

**Table 2:** Univariate and multivariate Cox proportional hazards regression models evaluating PNI, CONUT, and other risk factors for the entire patient cohort (n=237).

Analysis		Univariate			Multivariate		
Covariate		HR	95% CI	p-value	HR	95% CI	p-value
*CONUT*	*> 3 points*	2.1	1.6 – 2.9	**<0.001**	1.3	0.9 – 2.0	0.201
*PNI*	*≤ 36 points*	2.9	2.1 – 3.9	**<0.001**	1.9	1.2 – 3.0	**0.003**
*Age*	*≥ 70 years*	1.1	0.8 – 1.4	0.620			
*AFP*	*> 200 ng/ml*	1.3	1.0 – 1.8	0.081			
*Bilirubin level*	*≥ 1.2 mg/dl*	2.4	1.8 – 3.2	**<0.001**	1.9	1.4 – 2.6	**<0.001**
*AST level*	*> 31 U/L*	2.0	1.0 – 4.1	0.054			
*ALT level*	*≥ 35 U/L*	1.1	0.8 – 1.5	0.550			
*INR level*	*> 1.2*	1.2	0.9 – 1.7	0.260			
*Platelet count*	*> 150/nl*	1.3	0.9 – 1.8	0.140			
*Tumor number*	*≥ 2*	1.3	0.9 – 1.9	0.110			
*Max. lesion size*	*> 5.0 cm*	1.2	0.9 – 1.7	0.160			

Statistically significant p-values are depicted in bold.

In the subgroup of recommended TACE candidates, a high CONUT score, low PNI, and elevated bilirubin levels reached significance in univariate analyses. Multivariate Cox hazard regression for these factors showed significance for a low PNI and elevated bilirubin levels, whereas the CONUT score lost its predictive ability (**Table 3**).

**Table 3:** Univariate and multivariate Cox proportional hazards regression models evaluating the PNI, CONUT, and other risk factors for the subgroup of recommended TACE candidates (n=126).

Analysis		Univariate			Multivariate		
Covariate		HR	95% CI	p-value	HR	95% CI	p-value
*CONUT*	*> 7 points*	2.4	1.4 – 4.1	**0.001**	1.6	0.9 – 2.9	0.085
*PNI*	*≤ 39 points*	2.1	1.3 – 3.1	**<0.001**	1.6	1.1 – 2.5	**0.040**
*Age*	*≥ 70 years*	0.9	0.6 – 1.3	0.550			
*AFP*	*> 200 ng/ml*	1.0	0.6 – 1.5	0.910			
*Bilirubin level*	*≥ 1.2 mg/dl*	2.2	1.5 – 3.4	**<0.001**	1.8	1.1 – 2.8	**0.011**
*AST level*	*> 31 U/L*	1.2	0.5 – 2.7	0.690			
*ALT level*	*≥ 35 U/L*	1.1	0.7 – 1.7	0.600			
*INR level*	*> 1.2*	1.1	0.7 – 1.8	0.600			
*Platelet count*	*> 150/nl*	1.0	0.6 – 1.6	0.840			
*Max. lesion size*	*> 5.0 cm*	1.4	0.9 – 2.1	0.180			

Statistically significant p-values are depicted in bold.

As the CONUT score was not an independent risk factor for median OS, we aimed for a separate in-depth analysis of the individual parameters of both scores (**Table 4**). The optimal stratification cut-offs for the serum albumin level, total lymphocyte count, and serum cholesterol level were 32 g/L, 1234/mm^3^, and 211 mg/dl, respectively. A low serum albumin level (p<0.001) and low total lymphocyte count (p<0.001) both correlated with an impaired median OS in the univariate analysis. In the multivariate Cox regression model, both factors remained as independent predictors. Even when using optimal stratification, a low cholesterol level could not stratify patients according to their OS (p=0.260).

**Table 4:** Univariate and multivariate Cox proportional hazards regression models evaluating the individual PNI and CONUT score parameters.

Analysis	Univariate			Multivariate		
Covariate	HR	95% CI	p-value	HR	95% CI	p-value
*Serum albumin level* *≤32 g/L*	2.9	2.2 – 4.0	**<0.001**	2.9	2.1 – 3.9	**<0.001**
*Total lymphocyte count* *≤ 1234/mm^3^*	1.5	1.2 – 2.1	**0.004**	1.5	1.1 – 2.0	**0.011**
*Serum cholesterol level* *≤ 211 mg/dl*	1.2	0.9 – 1.8	0.260			

Statistically significant p-values are depicted in bold.

## Discussion

Both the PNI and CONUT score are easy-to-calculate immunonutritive scoring systems. While the PNI is calculated using serum albumin level and total lymphocyte count, the CONUT additionally includes the serum cholesterol level (19, 25). In this study, we performed the first head-to-head comparison of these two established immunonutritive scoring systems regarding their influence on OS in patients with HCC treated with TACE. Both scores showed some predictive ability in all patients, as well as in the subgroup of recommended TACE candidates. However, in multivariate Cox regression analysis, only the PNI remained a significant predictive factor. This is in accordance with cholesterol not being an independent predictive factor, whereas both serum albumin and lymphocyte count retained their predictive ability.

Inflammation has been identified as one of the potential key drivers for cancer development and progression (16, 17). Particularly for HCC development, inflammatory processes and counter-regulations play an essential role as HCC is a typical example of inflammation-linked cancer, as the vast majority of cases arise in injured liver tissue (17, 36, 37). Despite factors and pathways leading to non-resolving inflammation and transformation in unregulated proliferation, lymphocytes play an essential role in tumor defense, inhibiting cell migration and proliferation (16, 17, 36). Consequently, the measurement of systemic inflammation and changes in lymphocyte count may act as surrogate markers of changes in tumor behavior and the assessment of prognosis.

Immunonutritive scoring tries to combine both of the above-mentioned factors related to HCC development: The PNI incorporates albumin as a surrogate for impaired liver synthesis caused by liver tissue injury and lymphocyte count as an indicator of the immune response (19). The CONUT score comprises the same factors, but the nutritional component of the score is emphasized by the inclusion of cholesterol (25). Both scores may function as an addition to several established risk stratification models because they all failed external validation (11–13, 15, 38).

The PNI and CONUT score have been identified as independent prognostic factors for patients undergoing surgery or systemic treatment for HCC (21, 22, 31, 39). However, evidence is particularly lacking on immunonutritive scoring in patients treated with TACE and a head-to-head comparison of the scores is missing. To date, only two studies on both scores are available, and these were conducted in entirely different clinical settings: He et al. investigated the influence of the PNI as a risk factor for patients treated with TACE combined with recombinant human type-5 adenovirus H101 (24). In their study, the PNI was highly predictive in univariate analysis (p=0.001), and patients with a higher PNI had a superior survival in multivariate analysis (HR=0.685). However, the PNI did not reach significance (p=0.091). Similar results were observed by Liu et al. (23), who found that the PNI was a strong predictor of OS in univariate analysis. However, their study has statistical weaknesses: Even though patients with a high PNI had longer OS than patients with a low PNI in univariate analysis (p<0.001), they did not include this factor in their multivariate analysis, which is inconsistent with the statistical elucidations in their methods. Thus, evidence on the PNI in patients with HCC undergoing TACE is scarce. This especially pertains to Western patients, as both studies were conducted on Asian cohorts.

For the CONUT score, no studies are available on patients with HCC undergoing TACE. However, CONUT is a feasible stratification tool for patients undergoing surgery (30, 31). For locoregional treatment, two very recent studies have investigated the role of the CONUT score for Asian patients undergoing curative radiofrequency ablation (40, 41): Both groups were able to show that the CONUT score is an independent prognostic factor for OS. Nevertheless, we could not confirm these results in patients undergoing TACE, possibly due to the following reasons: First, in both studies, hepatitis B virus infection was the most common etiology. In our cohort, the most common etiology was alcohol, and the distribution of the etiological factors was more heterogeneous. Second, radiofrequency ablation is suggested for small tumors in patients with preserved liver function. This may lead to a more decisive influence of cholesterol as a surrogate marker for nutrition in these patients. In our study, TACE was applied to patients with intermediate or advanced stages of disease. In these patients, albumin is an essential factor for the overall outcome and functions as a surrogate marker of therapy tolerance (42–44). Thus, the albumin component may be superior and outweigh the nutritional aspect in these patients, which could be underrepresented in the factor weighting in the CONUT score. Third, in contrast to the above-mentioned studies, we compared the CONUT score to a different scoring system. Furthermore, with only two factors, instead of three, the PNI is easier to calculate and more cost-effective.

In terms of the scores’ calculation, one reason for the higher predictive ability of the PNI might be the fact that the PNI is calculated using continuous variables. Contrary to this, the CONUT is computed by converting continuous variables to rank data, which might lead to a loss of information.

Despite the evaluation of nutritional status using the PNI, the assessment of body composition parameters could have additive value in the evaluation of patients. Sarcopenia, which describes a quantitative and qualitative loss of skeletal muscle mass, has been identified as a strong prognostic factor for patients with HCC (45, 46). However, the association with the immunonutritive scoring is missing. Thus, our future goal is to compare and combine laboratory- and imaged-based body composition assessments for patients undergoing TACE.

In our study, bilirubin was strongly associated with poor survival. A combination of the PNI parameters serum albumin level and total lymphocyte count with the serum bilirubin level could probably lead to an even better stratification index. Future studies should validate this combination regarding its prognostic impact.

A problem of both scores that should also be addressed in future studies is the lack of defined cut-off values. To date, no reference values are available for the PNI and CONUT score. Therefore, the use of an optimal stratification method to determine cut-off values is justified. Nevertheless, even when using optimal stratification for our cohort’s best separation, the CONUT score did not reach significance in the multivariate analysis.

Clearly, the present study has several limitations. First, this study was conducted as a single center study. Second, the sample size was only moderate (n=237). One limiting factor for sample size may be the decision against imputing missing values. Only patients with complete datasets were included. Furthermore, we decided to only include patients from 2010 onwards to guarantee comparability of the procedure itself and the diagnosis and follow-up proceedings. However, compared to existing studies on this issue, the size was comparable. Third, we excluded patients who underwent subsequent curative therapies after TACE in order to avoid bias (47). Fourth, we did not perform any subgroup analysis of patients treated with different TACE techniques. However, multiple comparisons between cTACE and DEB-TACE have not shown any influence on OS (48–50). Furthermore, the latest evidence indicates that the utility of the scoring and staging systems did not differ with regards to the type of TACE (51).

## Conclusion

In general, immunonutritive scoring is promising for patients with HCC undergoing TACE. Both the PNI and CONUT score were able to stratify patients according to their median OS. However, taking into account additional established risk factors, only the PNI remained an independent prognostic factor; the CONUT score lost its predictive performance. Thus, PNI should be preferred when evaluating the nutritional status of patients undergoing TACE. However, before implementing the PNI as a scoring tool in the daily clinical routine, further validation studies are needed in different patient cohorts.

## Data Availability Statement

Data cannot be shared publicly because of institutional and national data policy restrictions im-posed by the Ethics committee of the Medical Association of Rhineland Palatinate, Mainz, Germany since the data contain potentially identifying patient information. Data are available upon request for researchers who meet the criteria for access to confidential data. Requests to access the datasets should be directed to roman.kloeckner@unimedizin-mainz.de.

## Ethics Statement

The studies involving human participants were reviewed and approved by Ethics committee of the Medical Association of Rhineland Palatinate, Mainz, Germany. Written informed consent for participation was not required for this study in accordance with the national legislation and the institutional requirements.

## Author Contributions

LM, FH, AM-K, FS, SG, FF, AW, PG, JM, DPDS, MP, CD, and RK devised the study, assisted in data collection, participated in the interpretation of the data, and helped draft the manuscript. LM, FH, FS, AM-K, and RK carried out the data collection. SG, FF, AW, PG, JM, MP, and CD supported the data collection efforts. LM, FH, DPDS, and RK created all of the figures and participated in the interpretation of data. LM, FH, DPDS, and RK performed the statistical analysis. All authors contributed to the article and approved the submitted version.

## Funding

LM, FS and SG are supported by the Clinician ScientistvFellowship “Else Kröner Research College: 2018_Kolleg.05”.

## Conflict of Interest

AW has received speaker fees and travel grants from Bayer. RK has received consultancy fees from Boston Scientific, Bristol-Myers Squibb, Guerbet, Roche, and SIRTEX and lectures fees from BTG, EISAI, Guerbet, Ipsen, Roche, Siemens, SIRTEX, MSD Sharp & Dohme.

The remaining authors declare that the research was conducted in the absence of any commercial or financial relationships that could be construed as a potential conflict of interest.
